# The preoperative prognostic value of the radiomics nomogram based on CT combined with machine learning in patients with intrahepatic cholangiocarcinoma

**DOI:** 10.1186/s12957-021-02162-0

**Published:** 2021-08-01

**Authors:** Youyin Tang, Tao Zhang, Xianghong Zhou, Yunuo Zhao, Hanyue Xu, Yichun Liu, Hang Wang, Zheyu Chen, Xuelei Ma

**Affiliations:** 1 Department of Liver Surgery, Liver Transplantation Center, West China Hospital of Sichuan University, No. 37 GuoXue Alley, Chengdu, 610041 People’s Republic of China; 2 West China School of Medicine, West China Hospital, Sichuan University, No. 37 GuoXue Alley, Chengdu, 610041 People’s Republic of China; 3 Department of Biotherapy, West China Hospital, Sichuan University, No. 37 GuoXue Alley, Chengdu, 610041 People’s Republic of China; 4 West China School of Public Health, NO.4 West China Teaching Hospital, Sichuan University, No. 18, three section of people south road, Chengdu, 610041 People’s Republic of China; 5 West China School of Medicine, West China Hospital, Sichuan University, No.14, 3Rd Section Of Ren Min Nan Rd., Chengdu, Sichuan 610041 People’s Republic of China; 6 Department of Liver Surgery, Division of Liver Transplantation Center, West China Hospital, Sichuan University, No. 37 GuoXue Alley, Chengdu, 610041 People’s Republic of China; 7 Department of Biotherapy, West China Hospital and State Key Laboratory of Biotherapy, West China Hospital, Sichuan University, No. 37 GuoXue Alley, Chengdu, 610041 People’s Republic of China

**Keywords:** Intrahepatic cholangiocarcinoma, Radiomics, Nomogram, Prognosis, Machine learning

## Abstract

**Background:**

Intrahepatic cholangiocarcinoma is an aggressive liver carcinoma with increasing incidence and mortality. A good auxiliary prognostic prediction tool is desperately needed for the development of treatment strategies. The purpose of this study was to explore the prognostic value of the radiomics nomogram based on enhanced CT in intrahepatic cholangiocarcinoma.

**Methods:**

In this retrospective study, 101 patients with pathological confirmation of intrahepatic cholangiocarcinoma were recruited. A radiomics nomogram was developed by radiomics score and independent clinical risk factors selecting from multivariate Cox regression. All patients were stratified as high risk and low risk by a nomogram. Model performance and clinical usefulness were assessed by calibration curve, ROC curve, and survival curve.

**Results:**

A total of 101patients (mean age, 58.2 years old; range 36–79 years old) were included in the study. The 1-year, 3-year, and 5-year overall survival rates were 49.5%, 26.6%, and 14.4%, respectively, with a median survival time of 12.2 months in the whole set. The least absolute shrinkage and selection operator (LASSO) method selected 3 features. Multivariate Cox analysis found three independent prognostic factors. The radiomics nomogram showed a significant prognosis value with overall survival. There was a significant difference in the 1-year and 3-year survival rates of stratified high-risk and low-risk patients in the whole set (30.4% vs. 56.4% and 13.0% vs. 30.6%, respectively, *p* = 0.018).

**Conclusions:**

This radiomics nomogram has potential application value in the preoperative prognostic prediction of intrahepatic cholangiocarcinoma and may facilitate in clinical decision-making.

**Supplementary Information:**

The online version contains supplementary material available at 10.1186/s12957-021-02162-0.

## Background

Intrahepatic cholangiocarcinoma (ICC) is an aggressive liver carcinoma, accounting for 10–15% of all primary liver malignancies [1–4]. In recent years, the incidence and mortality of ICC were increasing worldwide, albeit the incidence was still lower than hepatocellular carcinoma [5, 6]. Currently, surgical resection was still considered as the only curative treatment method for patients with resectable ICC [7, 8]. However, due to the insidious onset and fast progression of ICC, most patients presented with advanced stages when first diagnosed, with only 30 to 60% of ICC patients were able to receive surgery [9]. Even worse, the postoperative prognosis of patients who underwent “curative-intent” hepatectomy was disappointing [5]. The 5-year overall survival rate of ICC after surgical resection was up to 35% and that for advanced patients who failed to receive operation was falling short of 5% [10–14]. In addition, radiochemotherapy can only bring in a modest survival benefit for unresectable patients and was considered as an alternative method in reducing tumor burden and providing a second chance for surgical resection for patients with initially unresectable ICC [15–17]. Therefore, for patients with intrahepatic cholangiocarcinoma, a good auxiliary prognostic prediction tool is of great clinical value for the development of treatment strategies.

Texture analysis is a method to quantify texture parameters by qualitative analysis and calculation of intensity and spatial distribution characteristics of image pixels through post-processing of the traditional image [18–20]. At present, many researches have explored the value of texture analysis based on enhanced CT in tumor diagnosis and prognosis evaluation. In recent years, some studies have explored the ability of radiomics models to predict lymph node metastasis and early recurrence of ICC [21–23]. However, only few study investigated the preoperative prediction of long-term survival of ICC via radiomics, meaning that the clinical implementation value of radiomics signature in preoperative survival prediction of ICC was still worthy of attention. A nomogram predicts the probability of a clinical outcome by using the value of clinical indicators and the line segments marked by numbers through regression analysis [24]. The nomogram makes the results of the prediction model easier to understand through intuitive and simple graphics [25].

In the current study, we aim to construct and validate a comprehensive radiomics nomogram for predicting overall survival of ICC and identify high-risk patients who can only obtain a small survival benefit from hepatectomy.

## Methods

### Study population

We retrospectively reviewed an electronic medical record database of ICC patients treated at West China Hospital from October 2014 to July 2017. The including and excluding criteria were as follows: including criteria: (i) patients had complete medical record and follow-up, (ii) patients had definitely pathological diagnosis of ICC, (iii) patients had available enhanced CT scan within 2 weeks prior to surgery, (iv) patients were with Child-Pugh class A liver function and had no major vessel/bile duct infiltration or distant metastasis, and (v) patients had received a “curative-intent” surgical resection. The excluding criteria were as follows: (i) patients received any radiochemotherapy (including transcatheter arterial chemoembolization) before CT scan, (ii) patients only received exploratory operation (biopsy) or had distant metastasis of primary liver tumor, and (iii) patients had synchronous malignancy of other organs (co-malignancy).

**Fig. 1:**
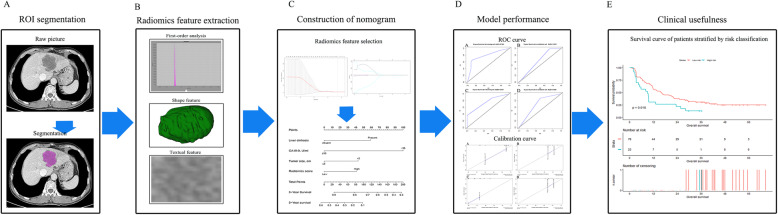
Study workflow. **a** ROI segmentation, **b** radiomics feature extraction and selection, **c** the procedure of construction of nomogram, **d** comparison of model performance, and **e** clinical decision analysis and survival comparison in the training set and validation set. OS, overall survival; ROI, region of interest; ROC, receiver operating characteristic

**Fig. 2:**
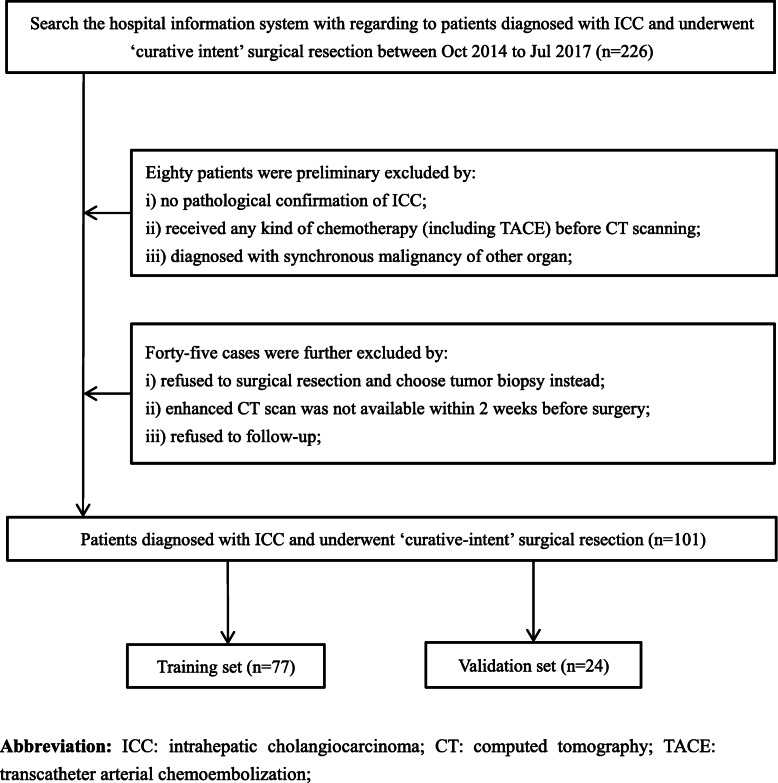
Patient selection flow diagram

Clinical data including 19 variables were recorded through the electronic medical record system. All CT data were obtained from the West China Hospital’s Radiology image database. Overall survival time was calculated from the date of surgery until either the date of death from any cause or until the date of the last follow-up. This study was approved by the ethics committee of West China Hospital of Sichuan University. Because this study did not involve the disclosure of any personal information, no written informed consent was required. The flow process of the whole study is shown in Fig. 1. The patient selection flow diagram is displayed in Fig. 2.

### CT techniques

Before the surgery, all patients underwent enhanced abdomen CT examinations by a single 64-detector row scanner (Brilliance 64, Philips Medical Systems, Eindhoven, the Netherlands). The uniform scan parameters are the following: beam pitch, 0.891; tube voltage, 120 kVp; tube current, 200 mAs; detector collimation, 0.75 mm; slice thickness, 1.0 mm; reconstruction increment, 5.0 mm; rotation time, 0.42 s; and matrix, 512 × 512. Computed tomography scanning was performed with 30 to 35 s for the arterial phase and 60 to 70 s for the portal venous phase [26, 27].

### Radiomics feature extraction and clinical factor selection

We retrieved CT digital imaging data from the West China Hospital’s imaging database. Tumor size and liver cirrhosis were recognized from preoperative CT scan by two well-experienced radiologists, and the differentiation grade of tumor was mainly obtained from preoperative fine-needle aspiration and postoperative pathological diagnosis. All CT data were selected for the portal venous phase. To quantify pathological lesion segmentation and automated quality features, we loaded them into the Local Image Features Extraction (LIFEx) software (v3.74, CEA-SHFJ, Orsay, France) for segmentation and texture analysis [28]. The region of interests (ROI) drawing process was completed by two radiologists independently. The ROIs were drawn freehand within the tumor lesion in the fusion images of enhanced CT. A total of 42 subdivided texture parameters were extracted (Supplementary table 1).

For all clinical variables, univariate analysis was performed for categorical variables and continuous variables, and then multivariate Cox analysis was adopted to search for potential independent survival risk factors.

### Radiomics nomogram

We divided the patients into training set and validation set according to the ratio of 3:1 randomly. In the training set, we used the least absolute shrinkage and selection operator (LASSO) Cox regression analysis to screen appropriate texture features which can predict the overall survival [29]. We linearly combined the selected texture features with their coefficients to create a radiomics score for each patient. The score corresponding to the maximum Youden coefficient in ROC results was defined as the threshold, and the radiomics scores of all patients in the training set and validation set were then stratified as high score and low score [30]. The nomogram was developed based on the dichotomous radiomics score and selected independent clinical parameters. In the nomogram, the score of each variable ranges from 0 to 100, while the variable with the highest hazard ratio is defined as 100 [20]. All patients were further classified into a high-risk group and a low-risk group from the nomogram. The performance of the nomogram was verified by calibration curve, ROC curve, survival curve, and decision curve analysis [31, 32].

### Statistical analysis

Continuous variables were displayed as mean ± standard deviation (SD), while categorical variables were expressed as frequency and percentage. The parametric test used Student’s *t* test whereas the nonparametric test used Mann-Whitney *U* test, *χ*2 test, and Fisher’s exact test. The Kaplan-Meier method and log-rank test were implemented to generate survival curves and compare the difference between groups. A two-sided *p* value < 0.05 was considered to denote statistical significance. All analyses were carried out with SPSS (V. 20.0 for Windows, IBM, Armonk, NY, USA) and R statistical software (V. 4.0.0, The R Foundation). The packages used in R software were glmnet, cmprsk, rms, survival, rmda, and devtools.

## Results

### Patients

A total of 101patients (mean age, 58.2 years old; range 36–79 years old) were included in this study. Among them, 55 patients were male and 46 patients were female. The 1-year, 3-year, and 5-year overall survival rates were 49.5%, 26.6%, and 14.4%, respectively, while the median survival time was 12.2 months in the whole set. In addition, the 1-year and 3-year overall survival rates of the training set were 49.4% and 30.9%, while those of the validation set were 50.0% and 12.5%, respectively. Except for aspartate aminotransferase (AST) level, no significant difference was noticed in baseline clinical characteristics between training and validation sets. The baseline characteristics of patients are shown in Table 1.

**Table 1:** The baseline characteristics of ICC patients in training and validation sets

Variable	Whole set (*n* = 101)	Training set (*n* = 77)	Validation set (*n* = 24)	*p*
Sex, male, *n* (%)	55 (54.5)	40 (51.9)	15 (62.5)	0.365
Age, mean ± SD, years	58.2 ± 10.8	58.5 ± 10.9	56.9 ± 10.7	0.458
Hypertension, *n* (%)				0.995
Yes	21 (20.8)	16 (20.8)	5 (20.8)	
No	80 (79.2)	61 (79.2)	19 (79.2)	
Diabetes, *n* (%)				0.932
Yes	8 (7.9)	6 (7.8)	2 (8.3)	
No	93 (92.1)	71 (92.2)	22 (91.7)	
Hepatitis B, *n* (%)	27 (26.7)	21 (27.3)	6 (25.0)	0.826
Liver cirrhosis, *n* (%)				0.074
Present	13 (12.9)	7 (9.1)	6 (25.0)	
Absent	88 (87.1)	70 (90.9)	18 (75.0)	
Hypersplenism, *n* (%)				0.421
Yes	2 (2.0)	1 (1.3)	1 (4.2)	
No	99 (98.0)	76 (98.7)	23 (95.8)	
ALT, mean ± SD, IU/L	36.0 ± 38.6	33.8 ± 37.3	43.3 ± 42.4	0.131
AST, mean ± SD, IU/L	38.7 ± 34.9	35.8 ± 34.4	48.6 ± 35.7	0.024
ALB, mean ± SD, g/L	41.6 ± 4.1	41.8 ± 3.7	41.2 ± 5.1	0.941
TBIL, mean ± SD, μmol/L	15.2 ± 16.5	13.6 ± 8.2	20.6 ± 30.7	0.095
PT, mean ± SD, s	11.7 ± 1.6	11.5 ± 1.0	12.3 ± 2.8	0.357
INR, mean ± SD	1.0 ± 0.1	1.0 ± 0.1	1.1 ± 0.2	0.362
AFP, mean ± SD, ng/mL	40.0 ± 189.7	29.1 ± 150.4	74.6 ± 283.6	0.624
CA 125, mean ± SD, U/mL	50.0 ± 82.8	47.3 ± 67.5	58.0 ± 120.9	0.106
CA 19-9, mean ± SD, U/mL	322.6 ± 393.5	321.2 ± 404.3	327.5 ± 365.4	0.515
CEA, mean ± SD, ng/mL	18.2 ± 71.1	13.0 ± 36.1	36.9 ± 137.6	0.333
Tumor size, *n* (%)				0.455
≤ 5 cm	27 (26.7)	22 (28.6)	5 (20.8)	
> 5 cm	74 (73.3)	55 (71.4)	19 (79.2)	
Differentiation				0.084
Well	1 (1.0)	0	1 (4.2)	
Moderate	36 (35.6)	31 (40.3)	5 (20.8)	
Poor	57 (56.4)	40 (51.9)	17 (70.8)	
Unclear	7 (6.9)	6 (7.8)	1 (4.2)	
OS, mean ± SD, m	19.9 ± 17.6	21.3 ± 18.6	15.6 ± 13.3	0.300

*Abbreviations*: *ICC* Intrahepatic cholangiocarcinoma, *SD* Standard deviation, *ALT* Alanine aminotransferase, *AST* Aspartate aminotransferase, *ALB* Albumin, *TBIL* Total bilirubin, *PT* Prothrombin time, *INR* International normalized ratio, *AFP* Alpha fetoprotein, *CA* Carbohydrate antigen, *CEA* Carcinoembryonic antigen, OS Overall survival

### Clinical prognostic factor selection

A total of 19 clinical variables were initially included and 5 of them with *p* value < 0.1 in univariate analysis were subsequently analyzed by multivariate analysis. Multivariate Cox analysis revealed that cirrhosis, CA19-9 level, and tumor size were independent clinical risk variables with *p* < 0.05. CA19-9 level (≥ 35 U/mL) had the highest hazard ratio (HR 3.984, 95% CI 2.146–7.407). These three clinical variables were considered as independent risk factors relating to OS of ICC. Univariate and multivariate analysis results are shown in Table 2.

**Table 2:** Univariate analysis and multivariate Cox regression analysis of clinical factors influencing overall survival outcomes in the training cohort

Variable	Univariate analysis, *p* value	Multivariate analysis, HR (95% CI)	*p* value
Sex, male	0.238		
Age, years	0.148		
Hypertension	0.804		
Diabetes	0.334		
Hepatitis B	**0.022**		0.324
Liver cirrhosis	**0.032**		**0.015**
Absent		ref	
Present		2.227 (1.169–4.242)	
Hypersplenism	0.888		
ALT, IU/L	0.582		
AST, IU/L	0.750		
ALB, g/L	0.650		
TBIL, μmol/L	0.831		
PT, s	0.247		
INR	0.480		
AFP level, ng/mL	0.696		
CA 125 level, U/mL	**0.083**		0.839
CA 19-9 level	**0.003**		**< 0.001**
< 35 U/mL		ref	
≥ 35 U/mL		3.984 (2.146–7.407)	
CEA level	0.172		
Tumor size	**0.007**		**0.006**
≤ 5 cm		ref	
> 5 cm		2.293 (1.263–4.167)	
Differentiation	0.409		

*Abbreviations*: *HR* Hazard ratio, *ALT* Alanine aminotransferase, *AST* Aspartate aminotransferase, *ALB* Albumin, *TBIL* Total bilirubin, *PT* Prothrombin time, *INR* International normalized ratio, *AFP* Alpha fetoprotein, *CA* Carbohydrate antigen, *CEA* Carcinoembryonic antigen, *ref* Reference

### Texture feature selection and radiomics score construction

Finally, from the 42 parameters extracted, we got a total of 7 feasible parameters. Through the least absolute shrinkage and selection operator (LASSO) Cox regression method, the lambda value of these parameters is 0.07283022 (Supplementary figures 1 and 2). These parameters and their coefficients are shown in Supplementary table 2. These parameters were combined linearly to get each patient’s radiomics score. The formula is shown in Supplementary table 3. According to the established formula, the radiomics scores of all patients in the training and validation sets were calculated. The threshold value of dichotomy of radiomics score was 1.2646 in the entire set.

### Construction and validation of the nomogram

**Fig. 3:**
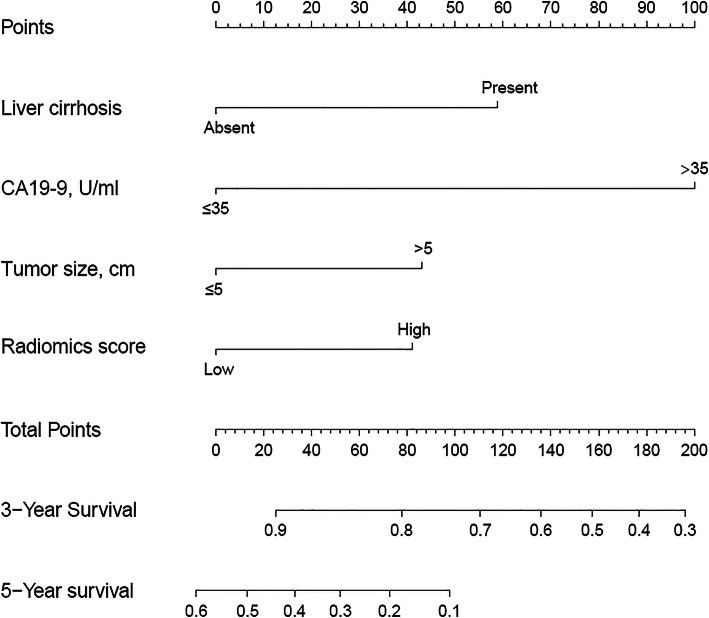
Nomogram for 3- and 5-year OS in patients with ICC

**Fig. 4:**
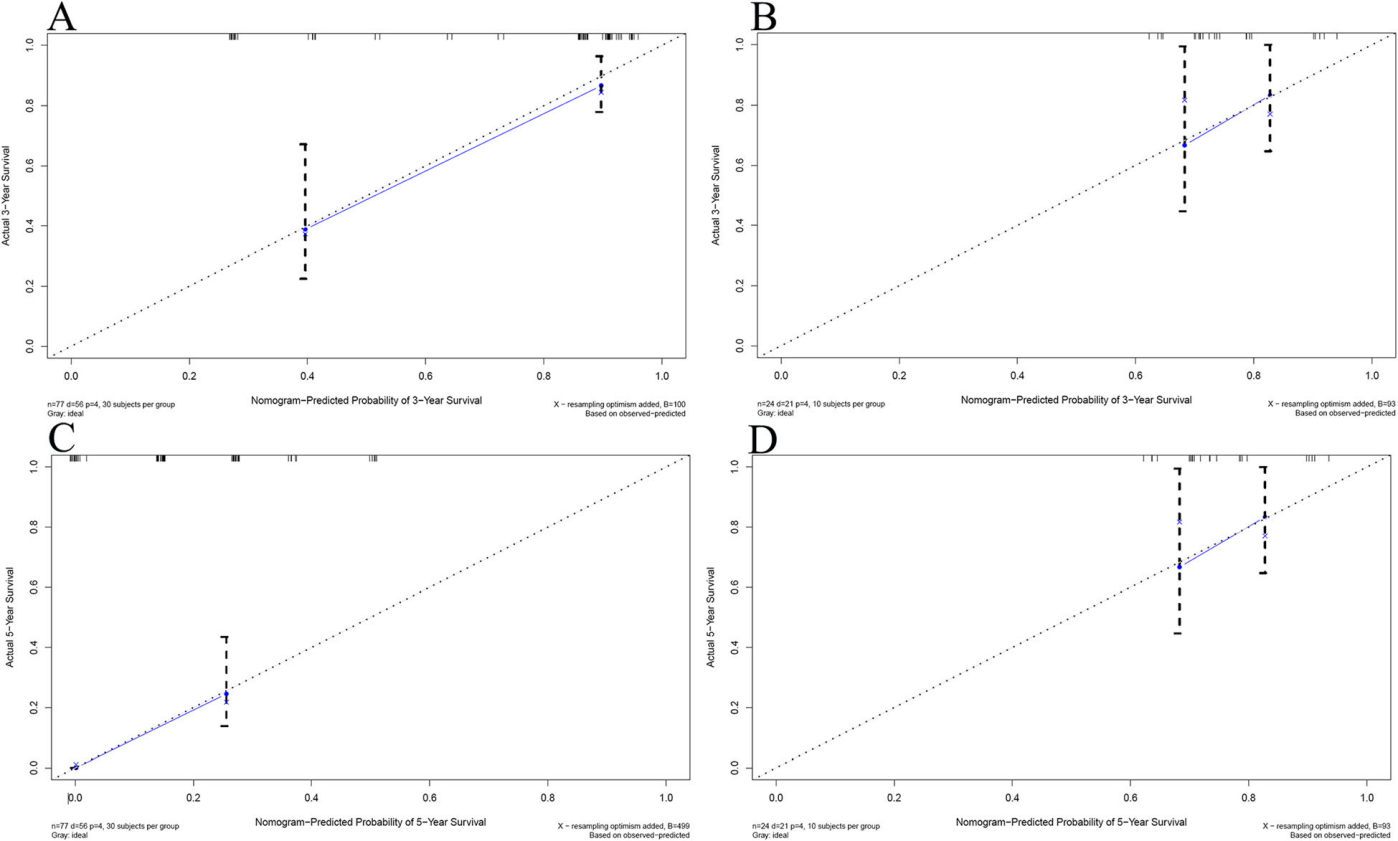
Calibration curves for overall survival (OS) at 3 years and 5 years in patients with ICC. **a** Three-year survival rate in the training set. **b** Three-year survival rate in the validation set. **c** Five-year survival rate in the training set. **d** Five-year survival rate in the validation set. The horizontal axis was the survival rate predicted by the nomogram, and the vertical axis was the actual survival rate. The dashed line indicates the predicting survival rate completely fits the actual survival rate. In the training set and validation set, the prediction results of the nomogram were close to the actual results of 3-year and 5-year OS, showing the calibration curve was in good agreement

**Fig. 5:**
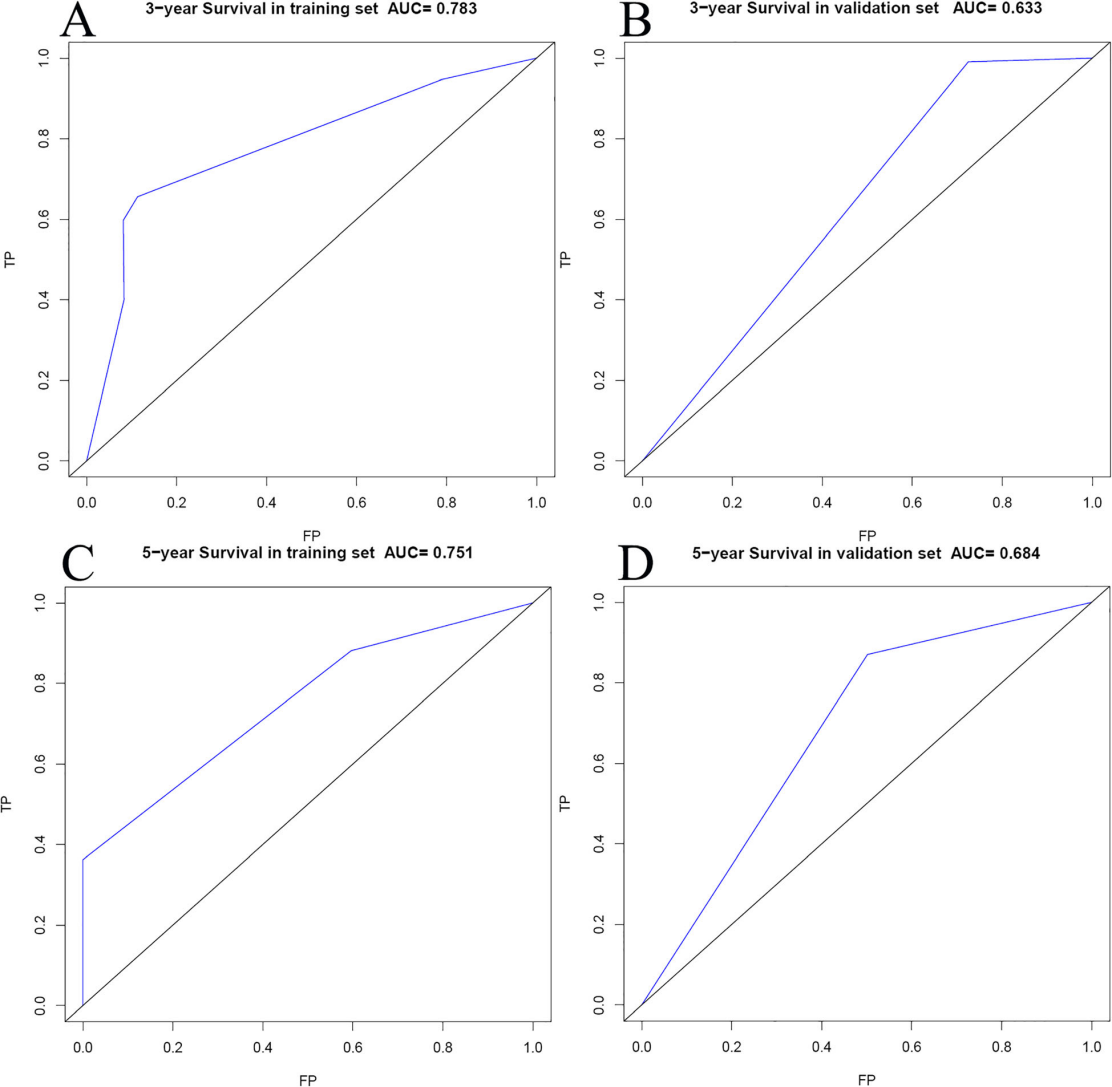
The ROC curves for overall survival (OS) at 3 years and 5 years in patients with ICC. a Three-year survival rate in the training set. **b** Three-year survival rate in the validation set. **c** Five-year survival rate in the training set. **d** Five-year survival rate in the validation set

Based on selected independent clinical predictors and radiomics score, we developed a comprehensive radiomics nomogram for preoperative predicting 3-year and 5-year overall survival rate in patients with ICC (Fig. 3). We applied the calibration curve (Fig. 4) and ROC curve (Fig. 5) to determine the predictive value and discriminative ability of the nomogram. In the training set and validation set, the prediction results of the nomogram were close to the actual results of 3-year and 5-year OS, showing the calibration curve was in good agreement. For the 3-year OS, the AUC was 0.783 in the training set and 0.633 in the validation set. For the 5-year OS, the AUC of the training set and validation set were 0.751 and 0.684, respectively.

### Risk stratification and prognosis comparison

**Fig. 6:**
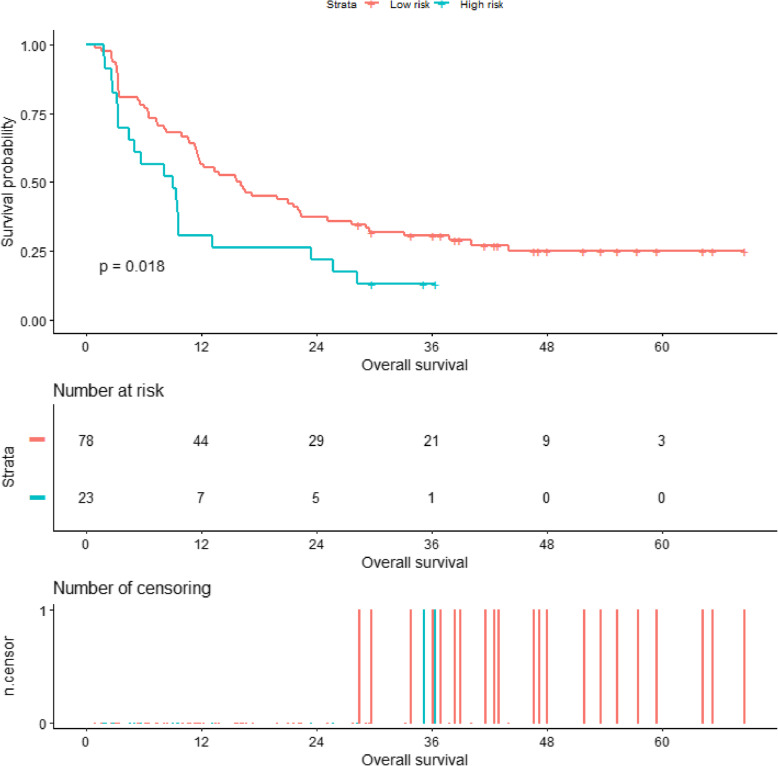
Overall survival rate of patients stratified by risk classification in the whole set. The results showed that the overall survival rate of high-risk patients was significantly lower than that of low-risk patients (*p* = 0.018)

**Fig. 7:**
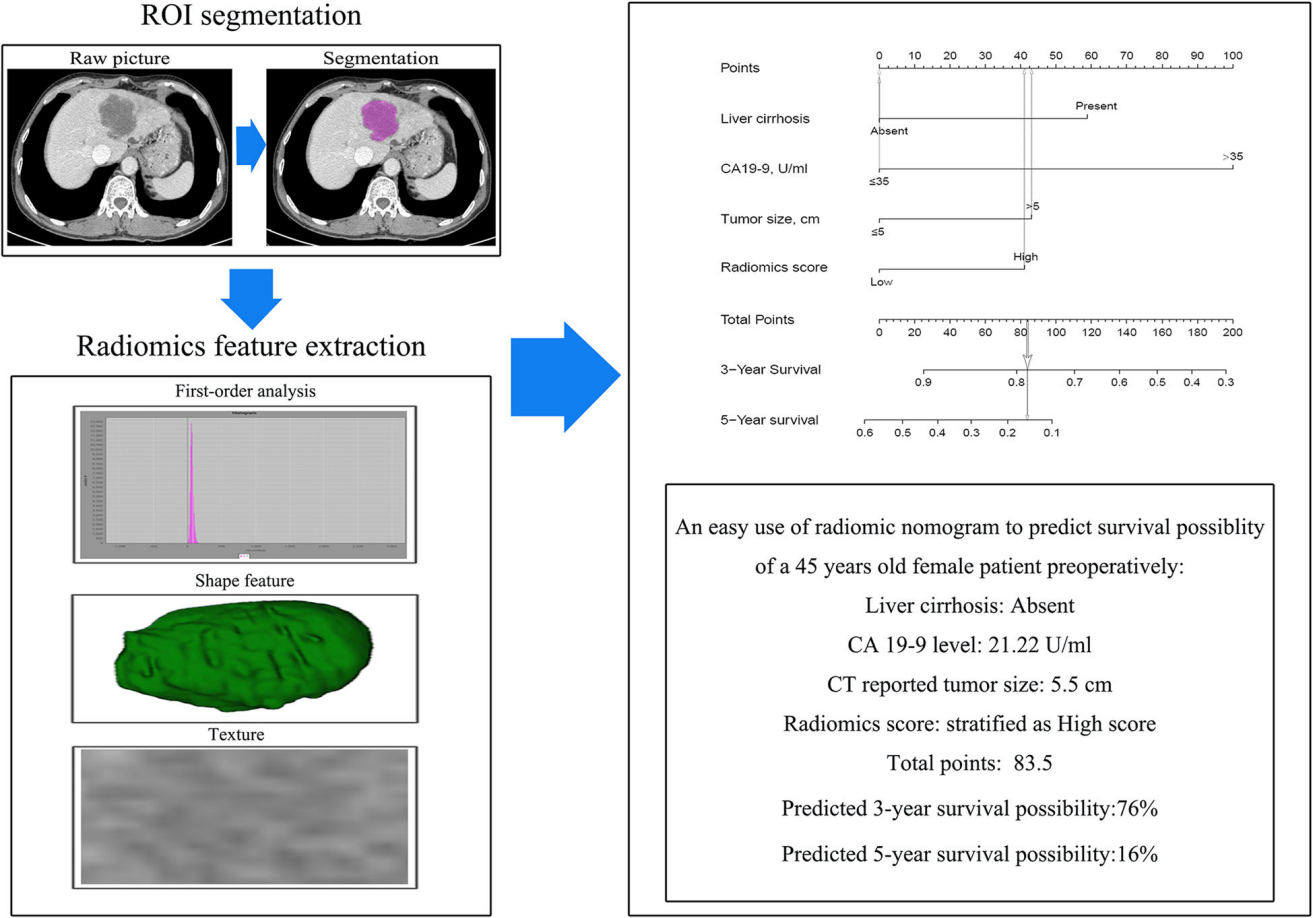
An example of using the radiomics nomogram to preoperatively predict overall survival probability in a 45-year-old female patient who underwent surgical resection

Add the scores of each variable to get the total nomogram score of each patient. According to the threshold (corresponding to 58.5 points of nomogram), all patients were further classified into a high-risk group and a low-risk group. Kaplan-Meier curve and log-rank test showed a significant difference in overall survival between high-risk and low-risk patients (3-year survival rates, high-risk versus low-risk 13.0% versus 30.6%, respectively, *p* = 0.018). The survival curve is shown in Fig. 6. An example of how to use the radiomics nomogram to preoperatively predict overall survival probability in a 45-year-old female patient is shown in Fig. 7. The radiomics model was built only by the selected radiomics signatures, and the clinical model was built only by clinical risk variables determined by multivariate analysis. The comprehensive radiomics nomogram was built by selected independent clinical predictors and radiomics score. And the decision curves of these three models are shown in Supplement Figure 3.

## Discussion

In the present study, we developed a comprehensive nomogram that contained enhanced CT-based radiomics parameters and clinical factors for preoperative survival prediction of ICC. And we found that radiomics score and clinical variables including tumor size, CA 19-9, and liver cirrhosis were all associated with overall survival. This study could be a supplementary of previous radiomics study, which only found CA 19-9 and radiomics score were associated with survival.

Texture analysis and prediction model based on pre-treatment-enhanced CT has potential value in prognosis prediction of patients with intrahepatic cholangiocarcinoma. At the same time, a latest review showed that the recurrence rate was high due to the fact that one-third of ICC patients were found to have lymph node metastasis, and the recurrence rate can be as high as 61–98% in 5 years. In this study, we found that patients who were stratified as high survival risk had a nearly 5-fold higher risk of death event than low-risk patients. In this study, we found that patients who were stratified as high survival risk suffered a significant worse prognosis than low-risk patients, with a median overall survival time of 9.0 and 15.9 months in high-risk patients and low-risk patients, respectively. These findings were in agreement with a previous radiomics study, which found that ICC patients with high risk of lymph node metastasis had a fivefold increased risk of death than low-risk patients [33]. Moreover, the conventional adjuvant radiochemotherapy could be used for high survival risk patients. Therefore, preoperatively predicting the survival of patients with ICC may facilitate in clinical decision-making.

Tumor size and CA 19-9 level had been widely used to evaluate the prognostic value of ICC for years. In the current study, we found that patients with elevated CA19-9 level or enlarged tumor size (> 5 cm) had an increased risk of death event of 4-fold and 2-fold than patients who presented with low CA19-9 level or small tumor size (≤ 5 cm), respectively. These findings were in agreement with several large series which also found that high serum CA19-9 and large tumor size were all independent risk factors relating to postoperative survival [7, 9, 12, 34]. In addition, previous studies found that elevated CA19-9 level was associated with a 1.62-fold increased death risk than normal patients and found that enlarged tumor size was associated to worse survival, with a hazard ratio of 1.09 [5, 8]. The rate of liver cirrhosis in the present study was 12.9% whereas the incidence of liver cirrhosis in ICC was reported to range between 9.5 and 12.3% [35, 36]. Moreover, a previous large-scale study found that the presence of liver cirrhosis had a tenfold increased risk of suffering from cholangiocarcinoma than no cirrhosis patients [37]. In this study, we found that the presence of liver cirrhosis was associated with poor survival, with a significantly increased death-even risk in the patient presented with cirrhosis (HR 2.227, 95% CI 1.169–4.242, *p* = 0.015). This was in agreement with a recently large multicenter study which revealed that liver cirrhosis was associated with very early recurrence (< 6 months) of ICC after surgical resection [35]. From the results, the patients in this study have some homogeneity, no matter in the ratio of men and women or the differentiation degree. The survival time obtained is also close to that reported in a previous study, with a reported 5-year post-hepatectomy survival rate range from 10 to 49% [11, 38, 39]. It is hopeful that the prognosis of low-risk patients was nearly 7 months longer than that of high-risk patients. The result was reflected from the training set and validation set, meaning that the radiomics nomogram may obtain potential value in preoperative prediction of prognosis of patients who underwent surgical resection.

CT radiomics was a noninvasive and cost-saving method in tumor identification and prognostic prediction, which obtained potential superiority than traditional fine-needle biopsy. In recent years, due to the rapid development of radiomics, CT texture features of tumor were gradually used to evaluate the prognosis of patients. At present, the most relevant application of textures in ICC was that many researchers use CT or MRI texture features to predict lymph node metastasis and early recurrence in patients with ICC, and the predicting value was high [23, 33, 40]. In this study, 7 texture parameters were finally included to preoperatively predict the overall survival of ICC patients.

In fact, these three parameters (PARAMS_ZSpatialResampling, PARAMS_YSpatialResampling, and PARAMS_XSpatialResampling) cannot be regarded as texture parameters strictly, but it was found in our study to be related to the survival time of patients. One possible explanation we think was that texture feature value can be significantly affected by voxel size, while voxel size is the standard value that can be set according to the coordinate system reference system, and then the obtained space resampling value should also have an impact on texture feature value [41–43]. The remaining four parameters were from gray-level co-occurrence matrix (GLCM) and gray-level run length matrix (GLRLM); they were real texture feature parameters. The GLCM considered the arrangements of voxel pairs to calculate textural indices, while the GLCM was calculated from thirteen different directions in three dimensions with a δ-voxel distance relationship between adjacent voxels. GLCM_Correlation was the linear dependency of gray levels in GLCM and GLCM_Dissimilarity was the variation of gray-level voxel pairs. The GLRLM gave the size of homogeneous runs for each gray level. In these parameters, Short-Run Low Gray-level Emphasis (SRLGE) revealed short homogeneous running distribution with low or high gray levels whereas the Gray-Level Non-Uniformity (GLNU) showed the non-uniformity running distribution of the gray levels or the length of the homogeneous runs [29, 43].

The two parameters from GLCM reflect the difference of tumor voxel size, which was expressed by the relative size and distance of voxels. The two parameters from GLRLM reflect the difference of tumor in gray scale, which was expressed by the uniformity of its distribution. The fundamental reason lies in the heterogeneity of tumor. We knew that the prognosis of the tumor was related not only to the malignancy of the tumor itself, but also to the basic health of the patients. The treatment measures they receive were closely related to their psychological factors. But it was undeniable that the most important factor was the tumor itself, especially its pathological manifestations.

We suspect that the prognosis of different patients was largely due to the pathological deterioration of the tumor itself. So the difference of microscopic cell expression of tumor with different pathological manifestations can be projected to the macroscopic level, which can be detected by imaging technology. This was also one of the theoretical bases of radiomics research in recent years [21, 22, 33, 35]. As far as we know, the direct relationship between the ICC aggressiveness and radiomics score remains unclear. Segal found that there was a correlation between CT radiomics features and the whole gene expression of hepatocellular carcinoma, in which they reconstructed 78% of the gene information through 28 CT radiomics features and then predicted the cell proliferation, liver synthesis function, and prognosis of patients with hepatocellular carcinoma [44]. And Chaisaingmongkol et al. found that ICC and HCC share repetitive mutated genes and similar actionable drivers, including TP53, ARID2, and ARID1A [45]. Therefore, the gene activity of liver cancer can be decoded noninvasively by imaging. In addition, in terms of histological classification, Wu found radiomics features were related to histological subtypes of lung cancer (adenocarcinoma and squamous cell carcinoma). They extracted 440 radiomics features from preoperative CT images and found 53 features were significantly correlated with tumor histology in univariate analysis and 5 most relevant features were finally selected to build a histological classification model through multivariate analysis [46]. In fact, there was a considerable amount of evidence to prove the value of GLCM and GLRLM in tumor prognosis evaluation, but the parameters found in different studies were not completely consistent [40, 47–49]. Zhao found that GLCM were related to early recurrence of ICC [40]. And although some single texture parameters were statistically correlated with survival time, the heterogeneity of tumor was influenced by multiple factors, so we considered that building a preoperative predictive radiomics-based model with multiple texture parameters may help in optimizing treatment outcomes and facilitating clinical decision-making of ICC. The nomogram showed the ability of distinguishing good prognosis from poor prognosis.

This study has some advantages. First of all, one of the advantages of this study is that compared with previous studies, this study involves a wider range of texture parameters, not only classic first-order parameters such as histogram, shape, etc., but also four types of texture parameters: GLCM, GLRLM, NGLDM, and GLZLM, which provides more research possibilities and innovation. Secondly, we have a long follow-up span and high reliability of survival data. In addition, it is particularly important that the enhanced CT examination process adopted by the patients we have included can ensure uniformity and eliminate the influence of certain confounding factors. Of course, the image post-processing in this study also achieved standardized operation to eliminate human interference. In addition to finding that these parameters are related to prognosis, we further established a prognostic scoring model based on imaging and obtained a threshold with a certain degree of credibility. The last advantage is that there is an internal validation set to improve the credibility.

Of course, this study also has some limitations. The first is that the number of patients is not large. In order to ensure the availability and homogeneity of the imaging data of the studied patients, the number of patients finally included in the study is not particularly large. Second, this study lacks external validation. However, we did an internal validation instead, and the AUC in the training set and validation set was well; for the 3-year OS, the AUC was 0.783 in the training set and 0.633 in the validation set; and for the 5-year OS, AUC of training and validation sets were 0.751 and 0.684, respectively.

## Conclusions

This radiomics nomogram based on texture analysis of preoperative enhanced CT has potential application value in the preoperative prognostic prediction of intrahepatic cholangiocarcinoma and may facilitate in clinical decision-making.

## Supplementary Information

**Additional file 1: **S**upplement Table 1.** Description of all texture features.

**Additional file 2: Supplement Table 2.** Selected texture features and their coefficients.

**Additional file 3: Supplement Table 3.** The formulas for constructing the radiomics score.

**Additional file 4: Supplement Figure 1 and 2.** Radiomics feature selection using a parametric method, the LASSO logistic regression.

**Additional file 5: Supplement Figure 3.** Decision curve analysis OS of radiomics score model, nomogram and clinical model in the training set. The y-axis measures the net benefit. The red line represents the nomogram. The blue dotted line represents the clinical model. The yellow dotted line represents the radiomics score model. The gray line represents the assumption that all patients dead. The black line represents the assumption that no patients dead.

## Data Availability

The data supporting the conclusion in this study can be obtained by contacting the corresponding authors.

## Abbreviations

ICC: Intrahepatic cholangiocarcinoma
ROI: The region of interests
HR: Hazard ratio
ALT: Alanine aminotransferase
AST: Aspartate aminotransferase
ALB: Albumin
TBIL: Total bilirubin
PT: Prothrombin time
INR: International normalized ratio
AFP: Alpha fetoprotein
CA: Carbohydrate antigen
CEA: Carcinoembryonic antigen

## References

[1] Torre LA, Bray F, Siegel RL, Ferlay J, Lortet-Tieulent J, Jemal A (2015). Global cancer statistics, 2012. CA Cancer J Clin.

[2] Kamarajah SK (2019). Evaluation of the AJCC 8th edition staging system for pathologically versus clinically staged intrahepatic cholangiocarcinoma (iCCA): a time to revisit a dogma? A Surveillance, Epidemiology, and End Results (SEER) analysis. J Gastrointest Cancer.

[3] Cheng R, Du Q, Ye J, Wang B, Chen Y (2019). Prognostic value of site-specific metastases for patients with advanced intrahepatic cholangiocarcinoma: a SEER database analysis. Medicine.

[4] Li JH, Zhu XX, Li FX, Huang CS, Huang XT, Wang JQ, Gao ZX, Li SJ, Xu QC, Zhao W, Yin XY (2019). MFAP5 facilitates the aggressiveness of intrahepatic cholangiocarcinoma by activating the Notch1 signaling pathway. Journal of experimental & clinical cancer research: CR.

[5] Mavros MN, Economopoulos KP, Alexiou VG, Pawlik TM (2014). Treatment and prognosis for patients with intrahepatic cholangiocarcinoma: systematic review and meta-analysis. JAMA surgery.

[6] Cidon EU (2016). Resectable cholangiocarcinoma: reviewing the role of adjuvant strategies, clinical medicine insights. Oncology.

[7] Wang Y, Li J, Xia Y, Gong R, Wang K, Yan Z, Wan X, Liu G, Wu D, Shi L, Lau W, Wu M, Shen F (2013). Prognostic nomogram for intrahepatic cholangiocarcinoma after partial hepatectomy. J Clin Oncol.

[8] Ribero D, Pinna AD, Guglielmi A, Ponti A, Nuzzo G, Giulini SM, Aldrighetti L, Calise F, Gerunda GE, Tomatis M, Amisano M, Berloco P, Torzilli G, Capussotti L (2012). Surgical approach for long-term survival of patients with intrahepatic cholangiocarcinoma: a multi-institutional analysis of 434 patients. Arch Surg.

[9] Endo I, Gonen M, Yopp AC, Dalal KM, Zhou Q, Klimstra D, D’Angelica M, DeMatteo RP, Fong Y, Schwartz L, Kemeny N, O’Reilly E, Abou-Alfa GK, Shimada H, Blumgart LH, Jarnagin WR (2008). Intrahepatic cholangiocarcinoma: rising frequency, improved survival, and determinants of outcome after resection. Ann Surg.

[10] Sonbare DJ (2014). Influence of surgical margins on outcome in patients with intrahepatic cholangiocarcinoma: a multicenter study by the AFC-IHCC-2009 Study Group. Annals of surgery.

[11] de Jong MC, Nathan H, Sotiropoulos GC, Paul A, Alexandrescu S, Marques H, Pulitano C, Barroso E, Clary BM, Aldrighetti L, Ferrone CR, Zhu AX, Bauer TW, Walters DM, Gamblin TC, Nguyen KT, Turley R, Popescu I, Hubert C, Meyer S, Schulick RD, Choti MA, Gigot JF, Mentha G, Pawlik TM (2011). Intrahepatic cholangiocarcinoma: an international multi-institutional analysis of prognostic factors and lymph node assessment. J Clin Oncol.

[12] Jiang W, Zeng ZC, Tang ZY, Fan J, Sun HC, Zhou J, Zeng MS, Zhang BH, Ji Y, Chen YX (2011). A prognostic scoring system based on clinical features of intrahepatic cholangiocarcinoma: the Fudan score. Ann Oncol.

[13] Bridgewater J, Galle PR, Khan SA, Llovet JM, Park JW, Patel T, Pawlik TM, Gores GJ (2014). Guidelines for the diagnosis and management of intrahepatic cholangiocarcinoma. J Hepatol.

[14] Macias RIR, Banales JM, Sangro B, Muntane J, Avila MA, Lozano E, Perugorria MJ, Padillo FJ, Bujanda L, Marin JJG (2018). The search for novel diagnostic and prognostic biomarkers in cholangiocarcinoma. Biochim Biophys Acta Mol Basis Dis.

[15] Riby D, Mazzotta A, Bergeat D, Verdure L, Sulpice L, Bourien H, Lièvre A, Rolland Y, Garin E, Boudjema K, Edeline J (2020). Downstaging with radioembolization or chemotherapy for initially unresectable intrahepatic cholangiocarcinoma. Ann Surg Oncol.

[16] Bargellini I, Mosconi C, Pizzi G, Lorenzoni G, Vivaldi C, Cappelli A, Vallati G, Boni G, Cappelli F, Paladini A, Sciuto R, Masi G, Golfieri R, Cioni R (2020). Yttrium-90 radioembolization in unresectable intrahepatic cholangiocarcinoma: results of a multicenter retrospective study. Cardiovasc Intervent Radiol.

[17] Cheng W, Liu Y, Zuo Z, Yin X, Jiang B, Chen D, Peng C, Yang J (2015). Biological effects of RNAi targeted inhibiting Tiam1 gene expression on cholangiocarcinoma cells. Int J Clin Exper Pathol.

[18] Castellano G, Bonilha L, Li LM, Cendes F (2004). Texture analysis of medical images. Clin Radiol.

[19] Lubner MG, Smith AD, Sandrasegaran K, Sahani DV, Pickhardt PJ (2017). CT texture analysis: definitions, applications, biologic correlates, and challenges. Radiographics.

[20] Miles KA, Ganeshan B, Hayball MP (2013). CT texture analysis using the filtration-histogram method: what do the measurements mean?. Cancer Imaging.

[21] King MJ, Hectors S, Lee KM, Omidele O, Babb JS, Schwartz M, Tabrizian P, Taouli B, Lewis S (2020). Outcomes assessment in intrahepatic cholangiocarcinoma using qualitative and quantitative imaging features. Cancer Imaging.

[22] Liang W, Xu L, Yang P, Zhang L, Wan D, Huang Q, Niu T, Chen F (2018). Novel nomogram for preoperative prediction of early recurrence in intrahepatic cholangiocarcinoma. Front Oncol.

[23] Xu L, Yang P, Liang W, Liu W, Wang W, Luo C, Wang J, Peng Z, Xing L, Huang M, Zheng S, Niu T (2019). A radiomics approach based on support vector machine using MR images for preoperative lymph node status evaluation in intrahepatic cholangiocarcinoma. Theranostics.

[24] Iasonos A, Schrag D, Raj GV, Panageas KS (2008). How to build and interpret a nomogram for cancer prognosis. J Clin Oncol.

[25] Balachandran VP, Gonen M, Smith JJ, DeMatteo RP (2015). Nomograms in oncology: more than meets the eye. Lancet Oncol.

[26] Zhou X, Luo Y, Peng YL, Cai W, Lu Q, Lin L, Sha XX, Li YZ, Zhu M (2011). Hepatic perfusion disorder associated with focal liver lesions: contrast-enhanced US patterns--correlation study with contrast-enhanced CT. Radiology.

[27] Zhao YJ, Chen WX, Wu DS, Zhang WY, Zheng LR (2016). Differentiation of mass-forming intrahepatic cholangiocarcinoma from poorly differentiated hepatocellular carcinoma: based on the multivariate analysis of contrast-enhanced computed tomography findings. Abdom Radiol (NY).

[28] Nioche C, Orlhac F, Boughdad S, Reuze S, Goya-Outi J, Robert C, Pellot-Barakat C, Soussan M, Frouin F, Buvat I (2018). LIFEx: A freeware for radiomic feature calculation in multimodality imaging to accelerate advances in the characterization of tumor heterogeneity. Cancer Res.

[29] Sauerbrei W, Royston P, Binder H (2007). Selection of important variables and determination of functional form for continuous predictors in multivariable model building. Stat Med.

[30] Youden WJ (1950). Index for rating diagnostic tests. Cancer.

[31] Fitzgerald M, Saville BR, Lewis RJ (2015). Decision curve analysis. Jama.

[32] Steyerberg EW, Vergouwe Y (2014). Towards better clinical prediction models: seven steps for development and an ABCD for validation. Eur Heart J.

[33] Ji GW, Zhu FP, Zhang YD, Liu XS, Wu FY, Wang K, Xia YX, Zhang YD, Jiang WJ, Li XC, Wang XH (2019). A radiomics approach to predict lymph node metastasis and clinical outcome of intrahepatic cholangiocarcinoma. Eur Radiol.

[34] Bagante F, Weiss M, Alexandrescu S, Marques H, Aldrighetti L, Maithel S, Pulitano C, Bauer T, Shen F, Poultsides G, Soubrane O, Martel G, Koerkamp B, Guglielmi A, Itaru E, Pawlik T (2018). Long-term outcomes of patients with intraductal growth sub-type of intrahepatic cholangiocarcinoma. HPB (Oxford).

[35] Tsilimigras DI, Sahara K, Wu L, Moris D, Bagante F, Guglielmi A, Aldrighetti L, Weiss M, Bauer TW, Alexandrescu S, Poultsides GA, Maithel SK, Marques HP, Martel G, Pulitano C, Shen F, Soubrane O, Koerkamp BG, Moro A, Sasaki K, Aucejo F, Zhang XF, Matsuyama R, Endo I, Pawlik TM (2020). Very early recurrence after liver resection for intrahepatic cholangiocarcinoma: considering alternative treatment approaches. JAMA Surg.

[36] Dhanasekaran R, Hemming AW, Zendejas I, George T, Nelson DR, Soldevila-Pico C, Firpi RJ, Morelli G, Clark V, Cabrera R (2013). Treatment outcomes and prognostic factors of intrahepatic cholangiocarcinoma. Oncol Rep.

[37] Sorensen HT, Friis S, Olsen JH, Thulstrup AM, Mellemkjaer L, Linet M, Trichopoulos D, Vilstrup H, Olsen J (1998). Risk of liver and other types of cancer in patients with cirrhosis: a nationwide cohort study in Denmark. Hepatology (Baltimore, Md.).

[38] Khan AS, Dageforde LA (2019). Cholangiocarcinoma. Surg Clin North Am.

[39] Conci S, Ruzzenente A, Viganò L, Ercolani G, Fontana A, Bagante F, Bertuzzo F, Dore A, Pinna AD, Torzilli G, Iacono C, Guglielmi A (2018). Patterns of distribution of hepatic nodules (single, satellites or multifocal) in intrahepatic cholangiocarcinoma: prognostic impact after surgery. Ann Surg Oncol.

[40] Zhao L, Ma X, Liang M, Li D, Ma P, Wang S, Wu Z, Zhao X (2019). Prediction for early recurrence of intrahepatic mass-forming cholangiocarcinoma: quantitative magnetic resonance imaging combined with prognostic immunohistochemical markers. Cancer Imag.

[41] Orlhac F, Theze B, Soussan M, Boisgard R, Buvat I (2016). Multiscale texture analysis: from 18F-FDG PET images to histologic images. J Nucl Med.

[42] Shafiq-Ul-Hassan M, Zhang GG, Latifi K, Ullah G, Hunt DC, Balagurunathan Y, Abdalah MA, Schabath MB, Goldgof DG, Mackin D, Court LE, Gillies RJ, Moros EG (2017). Intrinsic dependencies of CT radiomic features on voxel size and number of gray levels. Med Phys.

[43] Orlhac F, Nioche C, Soussan M, Buvat I (2017). Understanding changes in tumor texture indices in PET: a comparison between visual assessment and index values in simulated and patient data. J Nuclear Med.

[44] Segal E, Sirlin CB, Ooi C, Adler AS, Gollub J, Chen X, Chan BK, Matcuk GR, Barry CT, Chang HY, Kuo MD (2007). Decoding global gene expression programs in liver cancer by noninvasive imaging. Nat Biotechnol..

[45] Chaisaingmongkol J, Budhu A, Dang H, Rabibhadana S, Pupacdi B, Kwon SM, Forgues M, Pomyen Y, Bhudhisawasdi V, Lertprasertsuke N, Chotirosniramit A, Pairojkul C, Auewarakul CU, Sricharunrat T, Phornphutkul K, Sangrajrang S, Cam M, He P, Hewitt SM, Ylaya K, TIGER-LC Consortium (2017). Common molecular subtypes among Asian hepatocellular carcinoma and cholangiocarcinoma. Cancer Cell.

[46] Wu W, Parmar C, Grossmann P, Quackenbush J, Lambin P, Bussink J, Mak R, Aerts HJ (2016). Exploratory study to identify radiomics classifiers for lung cancer histology. Front Oncol..

[47] Wang M, Xu H, Xiao L, Song W, Zhu S, Ma X (2019). Prognostic value of functional parameters of (18)F-FDG-PET images in patients with primary renal/adrenal lymphoma. Contrast Med Mol Imaging.

[48] Kulkarni A, Carrion-Martinez I, Jiang NN, Puttagunta S, Ruo L, Meyers BM, Aziz T, van der Pol CB (2020). Hypovascular pancreas head adenocarcinoma: CT texture analysis for assessment of resection margin status and high-risk features. Eur Radiol.

[49] Brown PJ, Zhong J, Frood R, Currie S, Gilbert A, Appelt AL, Sebag-Montefiore D, Scarsbrook A (2019). Prediction of outcome in anal squamous cell carcinoma using radiomic feature analysis of pre-treatment FDG PET-CT. Eur J Nucl Med Mol Imaging.

