# Supplementary material for: The preoperative prognostic value of the radiomics nomogram based on CT combined with machine learning in patients with intrahepatic cholangiocarcinoma
Source: World J Surg Oncol. 2021 Aug 1;19:45. doi: 10.1186/s12957-021-02162-0 (PMC8327418; doi:10.1186/s12957-021-02162-0)
Supplement: Supplementary file 1 — Additional file 1: Supplement Table 1. Description of all texture features. [file 12957_2021_2162_MOESM1_ESM.pdf]

Supplementary table 1

Type of feature	Feature name	Description
Image-based parameters	minvalue	Describe its minimum
	meanvalue	Describe its mean
	stdvalue	Describe its standard deviation
	maxvalue	Describe its maximum
Co-occurrence matrix (GLCM): takes into account the arrangements of pairs of voxels to extract textural indices	Homogeneity	Homogeneity of grey-level voxel pairs
	Energy	Uniformity of grey-level voxel pairs.
	Correlation	Linear dependency of grey-levels in GLCM
	Contrast	Local variations in the GLCM
	Entropy	Randomness of grey-level voxel pairs
	Dissimilarity	Variation of grey-level voxel pairs
Grey-Level Run Length Matrix (GLRLM): gives the size of homogenous runs for each grey-level	SRE (short-run emphasis)	Distribution of the short homogeneous runs in an image
	LRE (long-run emphasis)	Distribution of the long homogeneous runs in an image
	LGRE (low grey-level run emphasis)	Distribution of the low grey-level runs
	HGRE (high grey-level run emphasis)	Distribution of the high grey-level runs
	SRLGE (short-run low grey-level emphasis)	Distribution of the short homogenous runs with low grey-levels
	SRHGE (short-run high grey-level emphasis)	Distribution of the short homogenous runs with high grey-levels
	LRLGE (long-run low grey-level Emphasis)	Distribution of the long homogeneous runs with low grey-levels
	LRHGE (long-run high grey-level emphasis)	Distribution of the long homogeneous runs with high grey-levels
	GLNU (grey-level non-uniformity)	Non-uniformity of the grey-levels of the homogeneous runs
	RLNU (run length non-uniformity)	Length of the homogeneous runs
	RP (run percentage)	Homogeneity of the homogeneous runs
Neighbourhood Grey-Level Different Matrix (NGLDM): corresponds to the difference of grey-level between one voxel and its 26 neighbourhoods in three	Coarseness	Level of spatial rate of change in intensity

dimensions	Contrast	Intensity diference between neighbouring regions
	Busyness	Spatial frequency of changes in intensity
Grey-Level Zone Length Matrix (GLZLM): provides information on the size of homogenous zones for each grey-level in three dimensions	SZE (short-zone emphasis)	Distribution of the short homogeneous zones in an image
	LZE (long-zone emphasis)	Distribution of the long homogeneous zones in an image
	LGZE (low grey-level zone emphasis)	Distribution of the low grey-level zones
	HGZE (high grey-level zone emphasis)	Distribution of the high grey-level zones
	SZLGE (short-zone low grey-level emphasis)	Distribution of the short homogenous zones with low grey-levels
	SZHGE (short-zone high grey-level emphasis)	Distribution of the short homogenous zones with high grey-levels
	LZLGE (long-zone low grey-level emphasis)	Distribution of the long homogeneous zones with low grey-levels
	LZHGE (long-zone high grey-level emphasis)	Distribution of the long homogeneous zones with high grey-levels
	GLNU (grey-level non-uniformity)	Non-uniformity of the grey-levels of the homogeneous zones
	ZLNU (zone length non-uniformity)	Length of the homogeneous runs
	ZP (zone percentage)	Homogeneity of the homogeneous zones
Indices from SHAPE: provides information from shape analysis	Sphericity	Measures how spherical a volume of interest is
	Compacity	Measures the degree to which the volume of interest is compact
	SHAPE_Volume (mL)	Measures the Volume of Interest in mL
	SHAPE_Volume (# vx)	Measures the Volume of Interest in voxels.
Indices from Histogram(HISTO): provides information derived from global histogram analysis	Skewness	Asymmetry of the grey-level distribution in the histogram.
	Kurtosis	Reflects the shape of the grey-level distribution (peaked or flat) relative to a normal distribution.
	Entropy	Measures the randomness of the distribution
	Energy	Measures the uniformity of the distribution
