# Supplementary material for: The preoperative prognostic value of the radiomics nomogram based on CT combined with machine learning in patients with intrahepatic cholangiocarcinoma
Source: World J Surg Oncol. 2021 Aug 1;19:45. doi: 10.1186/s12957-021-02162-0 (PMC8327418; doi:10.1186/s12957-021-02162-0)
Supplement: Supplementary file 2 — Additional file 2: Supplement Table 2. Selected texture features and their coefficients. [file 12957_2021_2162_MOESM2_ESM.pdf]

Table 2 Selected parameters

Parameter	Coefficient
PARAMS_ZSpatialResampling	-0.116560
PARAMS_YSpatialResampling	1.094233
PARAMS_XSpatialResampling	2.362806
GLCM_Correlation	1.255790
GLCM_Dissimilarity	-0.583620
GLRLM_SRLGE	-11457.340000
GLRLM_GLNU	0.000033
