# Supplementary material for: The preoperative prognostic value of the radiomics nomogram based on CT combined with machine learning in patients with intrahepatic cholangiocarcinoma
Source: World J Surg Oncol. 2021 Aug 1;19:45. doi: 10.1186/s12957-021-02162-0 (PMC8327418; doi:10.1186/s12957-021-02162-0)
Supplement: Supplementary file 3 — Additional file 3: Supplement Table 3. The formulas for constructing the radiomics score. [file 12957_2021_2162_MOESM3_ESM.pdf]

Table 3 The formulas for constructing the model

Model	Formula
Radiomics score	-0.116560PARAMS_ZSpatialResampling+1.094233PARAMS_YSpatialResampling+2.362806PARAMS_XSpatialResampling+1.255790GLCM_Correlation-0.583620GLCM_Dissimilarity-11457.340000GLRLM_SRLGE+0.000033GLRLM_GLNU
