# Supplementary figures and images for: The preoperative prognostic value of the radiomics nomogram based on CT combined with machine learning in patients with intrahepatic cholangiocarcinoma
Source: World J Surg Oncol. 2021 Aug 1;19:45. doi: 10.1186/s12957-021-02162-0 (PMC8327418; doi:10.1186/s12957-021-02162-0)

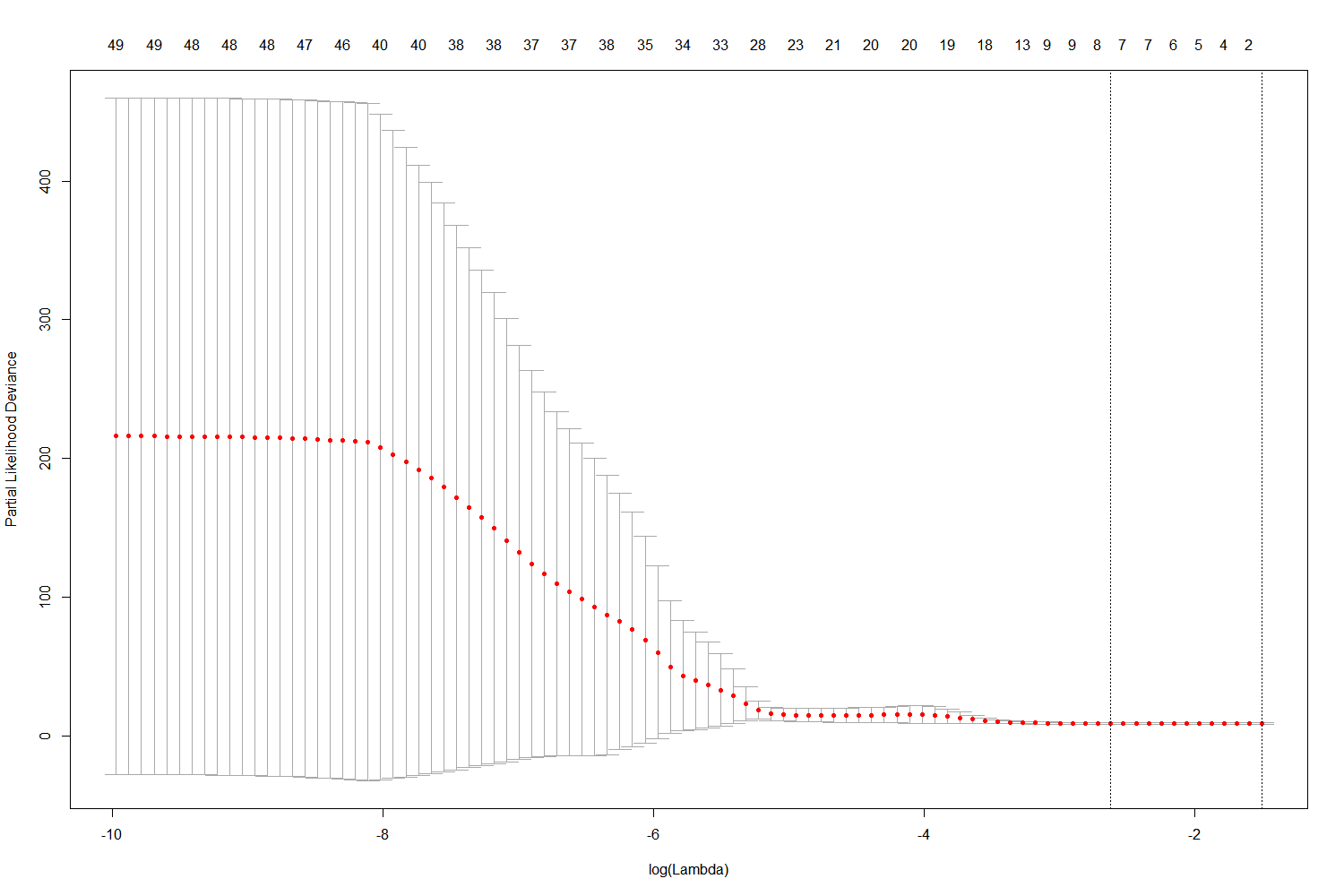

Supplement: Supplementary file 4 — Additional file 4: Supplement Figure 1 and 2. Radiomics feature selection using a parametric method, the LASSO logistic regression. [file 12957_2021_2162_MOESM4_ESM.zip › Supplementary figure 1.tiff]

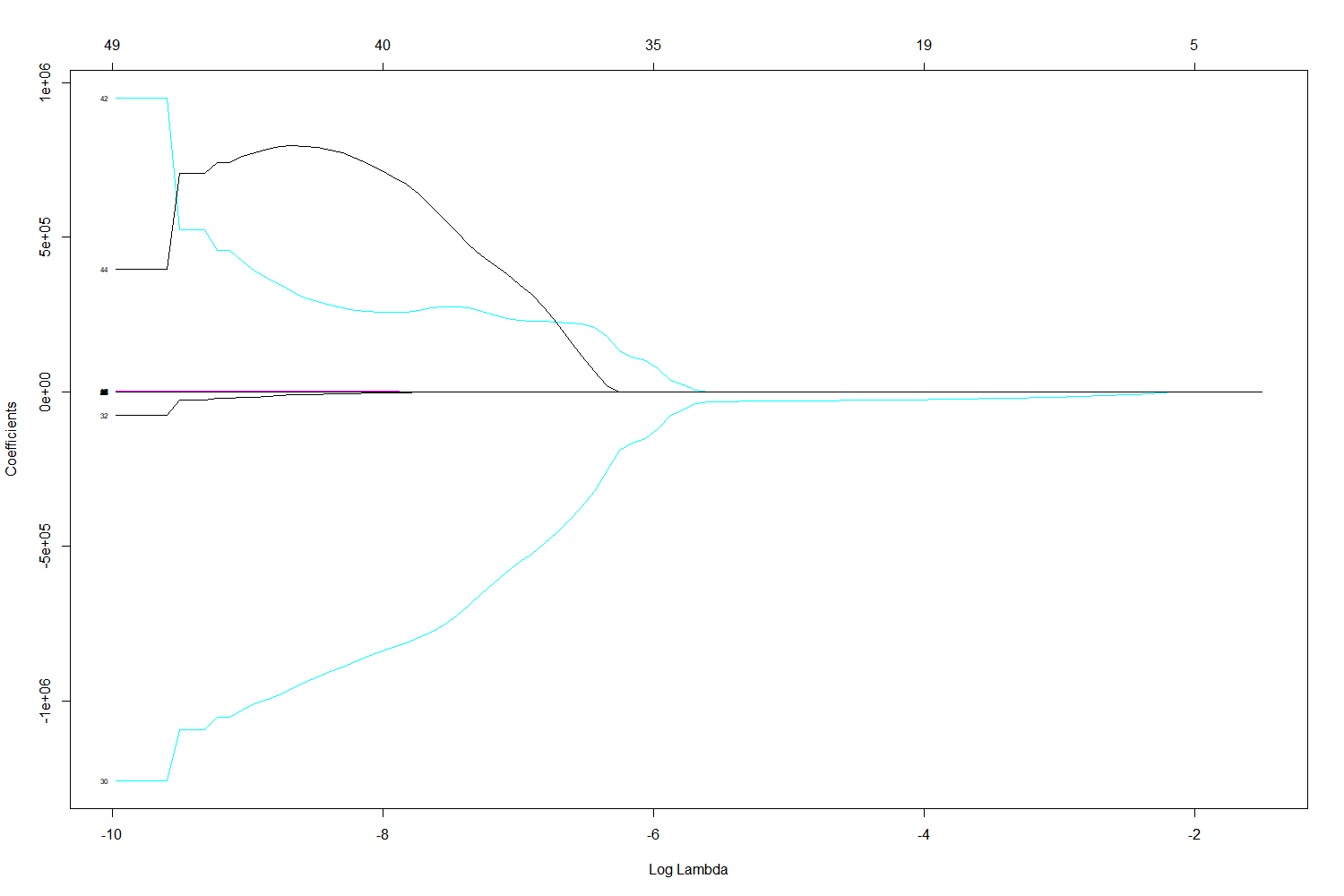

Supplement: Supplementary file 4 — Additional file 4: Supplement Figure 1 and 2. Radiomics feature selection using a parametric method, the LASSO logistic regression. [file 12957_2021_2162_MOESM4_ESM.zip › Supplementary figure 2.tiff]

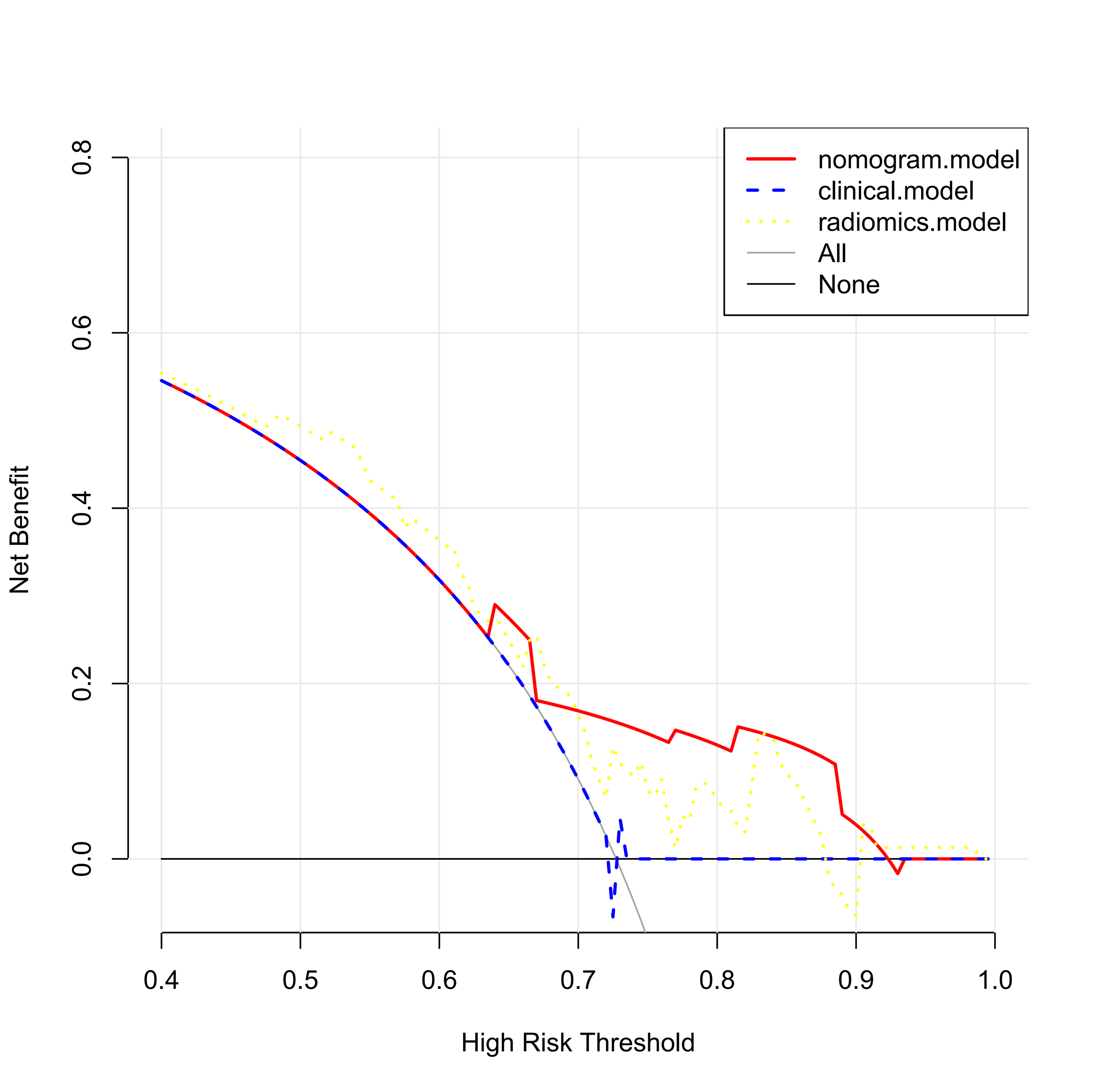

Supplement: Supplementary file 5 — Additional file 5: Supplement Figure 3. Decision curve analysis OS of radiomics score model, nomogram and clinical model in the training set. The y-axis measures the net benefit. The red line represents the nomogram. The blue dotted line represents the clinical model. The yellow dotted line represents the radiomics score model. The gray line represents the assumption that all patients dead. The black line represents the assumption that no patients dead. [file 12957_2021_2162_MOESM5_ESM.tif]
